# Supplementary material for: Quantum dot spin coherence governed by a strained nuclear environment
Source: Nat Commun. 2016 Sep 12;7:12745. doi: 10.1038/ncomms12745 (PMC5027245; doi:10.1038/ncomms12745)
Supplement: Supplementary Information — Supplementary Figures 1-6, Supplementary Tables 1-2, Supplementary Notes 1-3 and Supplementary References [file ncomms12745-s1.pdf]

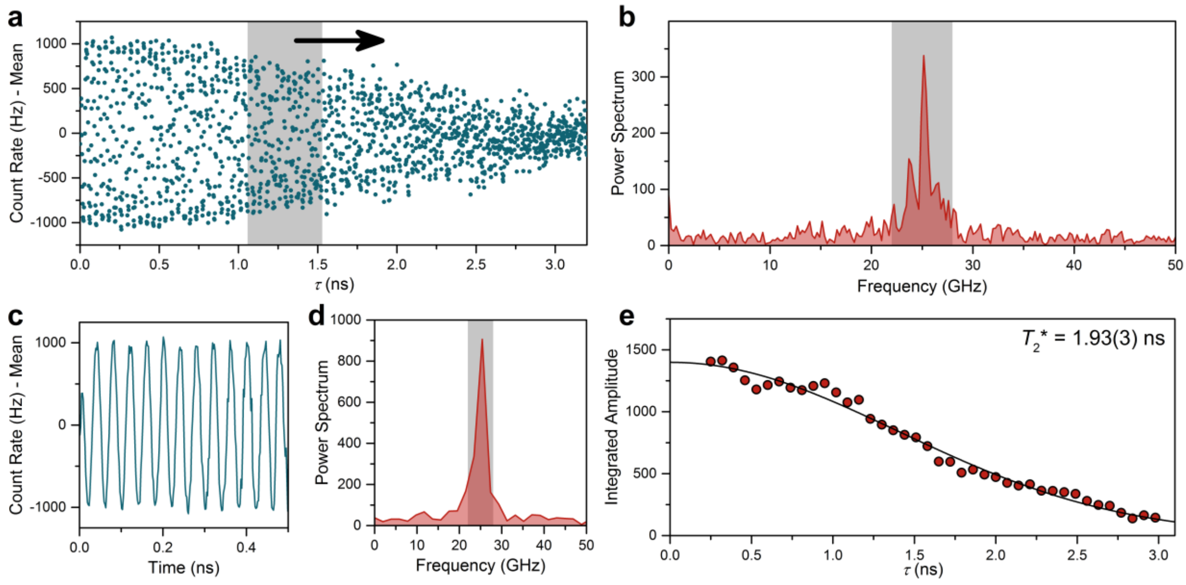

Supplementary Figure 1 **Analysis of free induction decay** (a) Free-induction decay count rates with mean count rate subtracted. (b) The Fourier transform of the decay shows a peak at 25 GHz, corresponding to the electron spin Larmor precession. (c) The first 0.5-ns window of data. (d) Fourier transform of the first window. The shaded area corresponds to the spectral band used for integrating the power. (e) Integrated spectrum values and corresponding fit, revealing a Gaussian decay with T_2* = 1.93(3) ns.

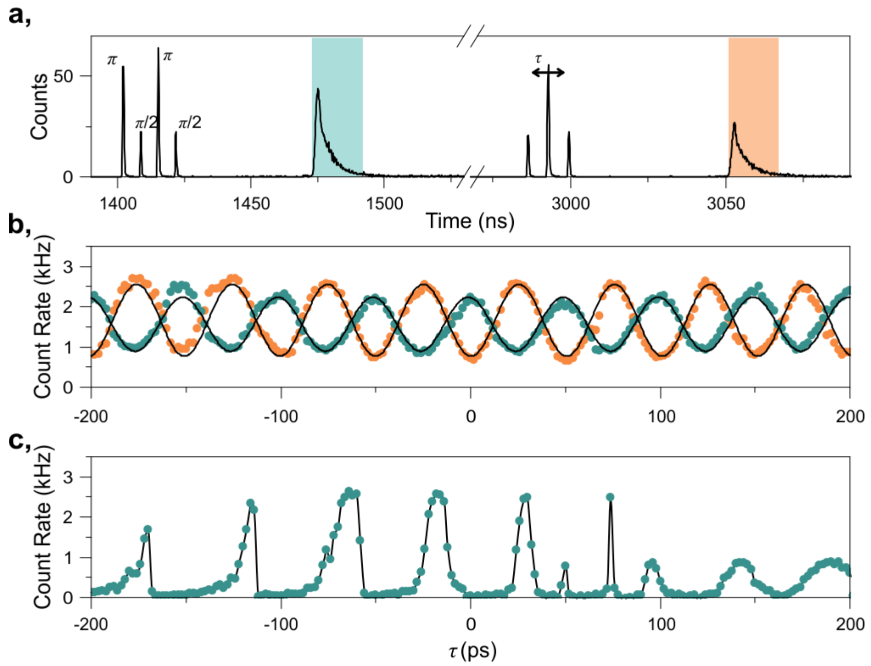

Supplementary Figure 2 **Dynamic nuclear spin polarisation in Hahn echo sequence** (a) Pulse sequence for 13.13 ns Hahn echo, highlighting readout regions of interest. Data taken at external field of 1.5 T. (b) Integrated readout count rates with (blue circles) and without (orange circles) initial inversion. Black continuous curves are sinusoidal fits to the data. (c) Corresponding trace measured in the absence of the alternating inversion pulse. The polarisation of the nuclear spin bath prevents readout of state coherence.

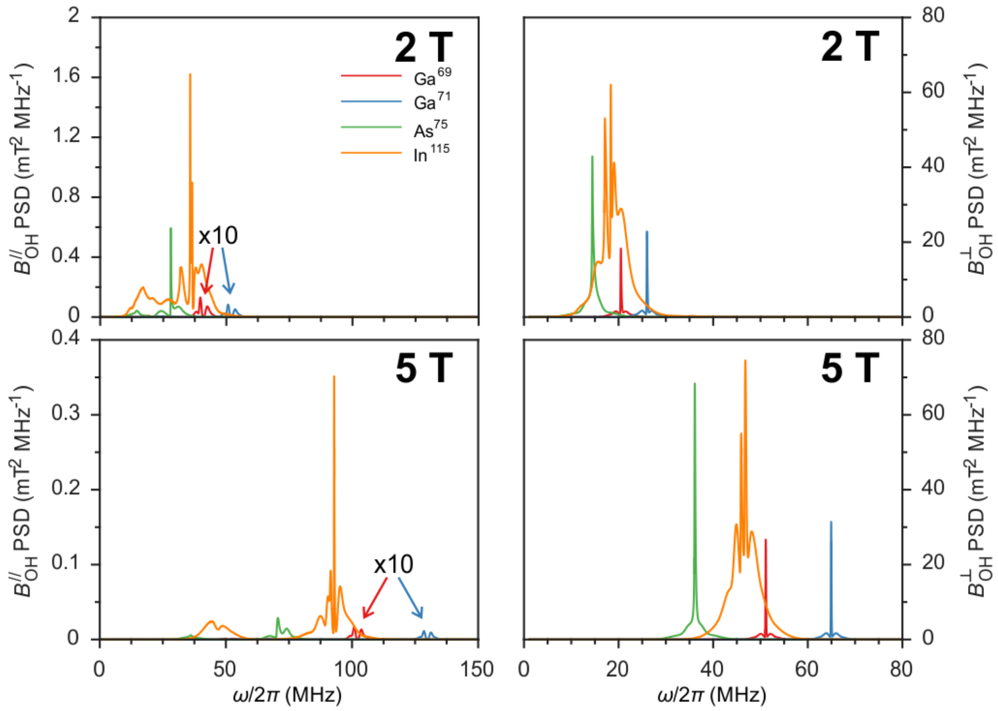

Supplementary Figure 3 **Spectra of nuclear spin species** The panels on the left-hand side show the parallel Overhauser field components, while the right hand-side panels display the perpendicular components. The dominance of indium nuclear spins is visible. For the parallel components the interplay of quadrupolar and Zeeman terms enables transitions between Zeeman eigenstates separates by two units of angular momentum.

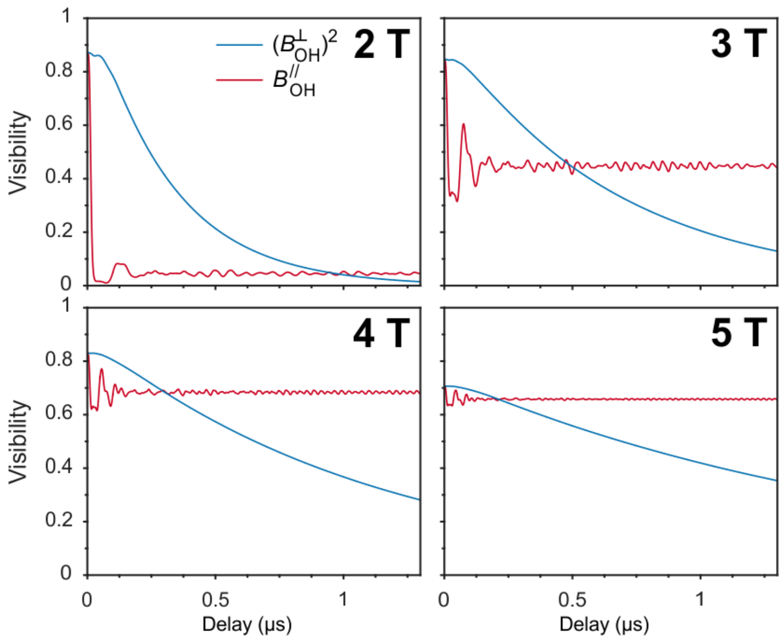

Supplementary Figure 4 **Deconstructed Hahn-echo functions** Red curves: Calculated Hahn-echo visibility when only the first-order coupling to the parallel Overhauser field components is taken into account. Blue curves: Calculated Hahn-echo visibility when only the second-order coupling to perpendicular Overhauser field components is considered.

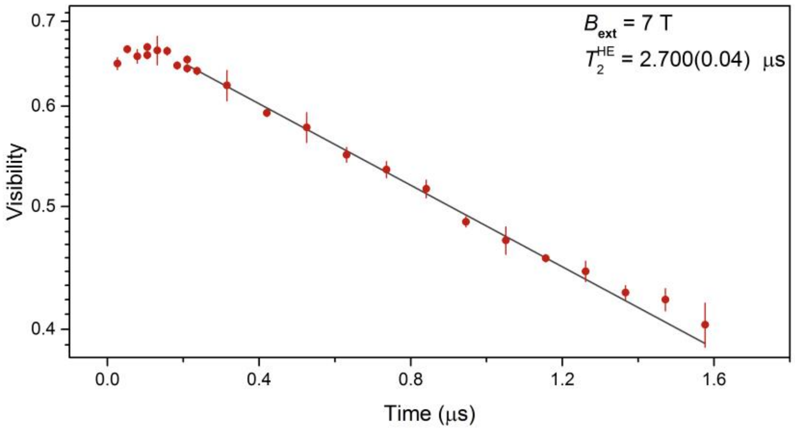

Supplementary Figure 5 **Hahn-echo visibilities recorded at 7 T for a different quantum dot** At this field, the short-time oscillations are strongly suppressed due to the Zeeman-dominated dynamics of the nuclear spin bath. We find a long-time decay with a characteristic decay constant of 2.7 µs, consistent with the suppression of quadratically coupled components of the nuclear bath at high fields. The decay timescale is consistent with our calculations for the quantum dot in the main text. Error bars show the standard error of the mean for repeated measurements.

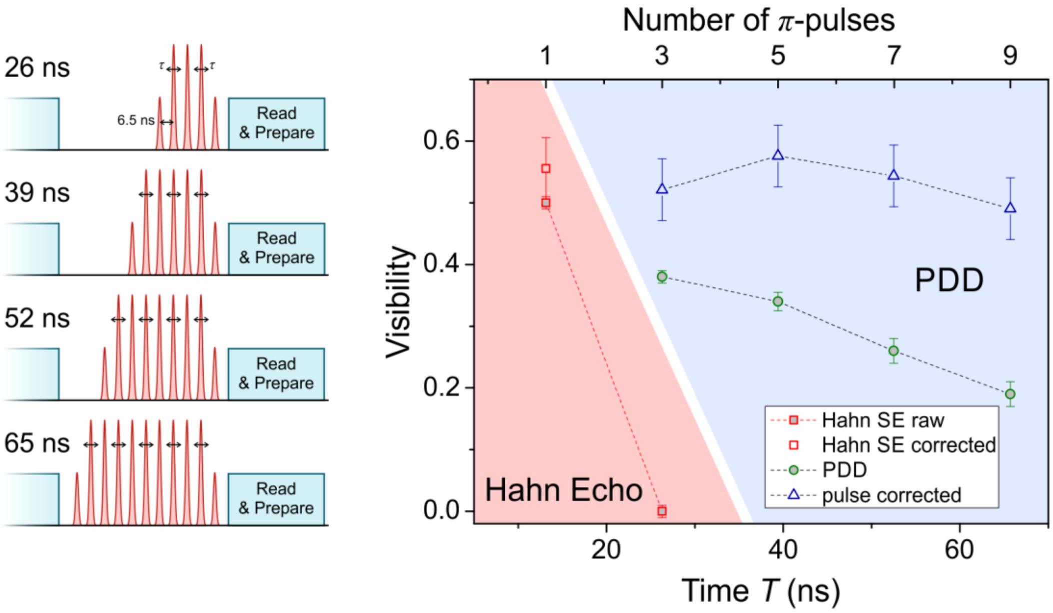

Supplementary Figure 6 **Periodic dynamic decoupling of electron spin coherence at 1.5 T external field** Left: Adding $2 \times n\ \pi$ pulses separated by 6.5 ns to the Hahn echo extends the sequence by $n \times 13.14$ ns. The readout pulse at the end provides a measurement of the coherence. Sweeping alternate π pulses measures the protected visibility. Right: Data in the red shaded area display results using the Hahn-echo sequence. For waiting times $T \geq 2 \times 13.14\ ns$, spin coherence cannot be recovered with a single inversion pulse, implying complete dephasing has taken place. Data in the area shaded in blue are measured using the PDD sequence for up to 9 π pulses (65 ns). Raw data is displayed as filled symbols, open symbols are obtained by accounting for the imperfect rotation angle. The error bars are the uncertainty in the fitted visibility. The extension of spin coherence beyond the Hahn-echo limit demonstrate dynamical decoupling is feasible using closely-spaced pulse sequences.

	I_k	A_k (μeV)	x_k	$g_k\mu_N$ (MHz/T)
**^{115}In & ^{113}In**	9/2	55	x	9.33
**^{69}Ga**	3/2	37	$0.6 \times (1-x)$	10.22
**^{71}Ga**	3/2	48	$0.4 \times (1-x)$	12.98
**^{75}As**	3/2	43	1	7.22

Supplementary Table 1 **Nuclear spin quantum numbers (I_k), hyperfine constants (A_k), concentrations (x_k) and Zeeman energies ($g_k\mu_N$) for the nuclear species present in the quantum dot.**

	^{115}In & ^{113}In	^{75}As	^{69}Ga	^{71}Ga
$\boldsymbol{\eta}$	0.315	0.5	0.358	0.358
$\boldsymbol{\nu_Q}$ (MHz)	3	5	3.3	3.3
$\boldsymbol{\sigma_{\nu_Q}}$ (MHz)	0.9	3	1.65	1.65
$\boldsymbol{\theta_{k,1}}$	15°	20°	11.3°	11.3°
$\boldsymbol{\sigma_{\theta_{k,1}}}$	5°	10°	5°	5°
$\boldsymbol{\theta_{k,2}}$	52.5°	-	-	-
$\boldsymbol{\sigma_{\theta_{k,2}}}$	12°	-	-	-

Supplementary Table 2 **Parameters describing the quadrupolar broadening of nuclear spin species for an indium concentration of $x \sim 0.5$. Included are the biaxiality parameter (η), the quadrupolar energy (ν_Q) and the width of its distribution ($\sigma_{\nu Q}$), the tilt of the quadrupolar axis (ϑ_k) and its corresponding width ($\sigma_{\vartheta k}$).**

Supplementary Note 1: Free induction decay

To find the timescale of the free induction decay (cf. Fig. 2 in the main text) and determine its functional form, we employ a moving Fourier transform method. The data from Fig. 2d is reproduced in Supplementary Figure 1a with the mean count rate subtracted. The power spectrum of the free induction decay is found by taking the fast Fourier transform (FFT) of the data, which is displayed in Supplementary Figure 1b. As expected, the spectrum contains the beat note at ~25 GHz corresponding to the electron splitting for an external in-plane field of 4 T.

We start by taking the FFT of the first 0.5 ns of data. The experimental data and resulting transform are displayed in Supplementary Figure 1c and d. We then sweep this window in 50-ps steps and find the integrated power within a 6-GHz window centred at 25 GHz. The integrated power as a function of window delay is plotted in Supplementary Figure 1e. Fitting to $\boldsymbol{A}\,\boldsymbol{e}^{-(\boldsymbol{T}/\boldsymbol{T}_2^*)^{\boldsymbol{\alpha}}}$ gives α = 2.03(0.11), consistent with a Gaussian decay, and a decay time of 1.96(3) ns. The scanning-window method introduces a slight overshoot in the decay time, which if corrected for reveals a $\boldsymbol{T}_2^*$ of 1.93(3) ns.

Supplementary Note 2: Suppression of bath polarisation in Hahn echo

We employ the same alternating pulse sequence used for free induction decay measurements to measure the Hahn echo of the electron spin. This is necessary to cancel the phase dependence in the average signal and suppress the build-up of nuclear spin polarisation. In order to show this, we repeat the spin-echo measurement, however this time without flipping the initial spin state every second measurement. The comparison of the two methods is displayed in Supplementary Figure 2. In the presence of the alternating flipping pulse (cf. Supplementary Figure 2b) the expected sinusoidal oscillatory behaviour can be observed when the position of the central pulse is swept, allowing the recall of the spin coherence. Removing the flipping pulse (cf. Supplementary Figure 2b) the state-dependent polarisation of the bath prevents the measurement of spin coherence.

Supplementary Note 3: Hahn-echo modelling

We calculate the evolution of the electron spin Hahn echo under the assumption that the Overhauser field can be treated as a classical effective field. The Hahn echo is calculated by introducing a noise spectrum and a Hahn-echo filter function following the approach taken in Cywinski *et al.*[1]. The noise spectrum determining the electron decoherence in our quantum dot is governed by the Larmor precession of the nuclear spin bath which is distorted and broadened by inhomogeneous strain-induced quadrupolar fields. In order to derive a noise spectrum for the effective Overhauser field we calculate the quantum evolution of a nucleus under a Hamiltonian which contains Zeeman and quadrupolar terms. We then sum over an ensemble of quadrupolar configurations and the different nuclear isotopes. The model is described in detail below.

Free induction decay and Overhauser field variance

In the free induction decay (FID) measurement presented Fig. 2 of the main manuscript, we observe the well-understood inhomogeneous dephasing (T_{FID}) of the elecstron spin due to slowly varying nuclear field fluctuations $\boldsymbol{B}_{\mathrm{OH}}$. This inhomogeneous dephasing can be related to the Overhauser field's standard deviation $\sigma(\boldsymbol{B}_{\mathrm{OH}})$:

$$T_{\mathrm{FID}} = \frac{\sqrt{6}\hbar}{\left(g_e \mu_B \sigma(\boldsymbol{B}_{\mathrm{OH}})\right)}, \tag{1}$$

where T_{FID} is taken at the $1/e$ point of the Gaussian FID decay. From $T_{\mathrm{FID}} = 1.93$ ns we estimate that the Overhauser field's standard deviation is 33 mT which is typical for this system.

The Overhauser field variance can be related to a number of nuclei (N) interacting with the electron spin. This number is arbitrary in the sense that it depends on a choice for the electronic envelope wave-function, however it provides a synthetic way of normalizing the spectra in the model and will therefore be used later on. Throughout this work, we assume two atoms per unit cell (volume v_0) and a flat electronic probability density in the quantum dot $\left(|\psi_{\mathrm{e}}|^2 = 2/(N v_0)\right)$. If we neglect correlations between nuclei the variance of nuclear fluctuations becomes

$$\sigma^2(\boldsymbol{B}_{OH}) = \frac{2}{\left(g_e\mu_B\sqrt{N}\right)^2} \sum_k x_k A_k{}^2 I_k(I_k+1), \tag{2}$$

with x_k, A_k, I_k representing the concentration, the hyperfine coupling constant and the spin of the nuclear isotope k. The values we take for x_k, A_k, $I_k$are presented in Supplementary Table 1.

Nuclear precession in the presence of quadrupolar interactions

If we neglect pairwise interactions between nuclei and hyperfine coupling to the electron spin the evolution of a single nucleus is simply given by the Hamiltonian

$$\hat{H}^j_{\text{nuc}} = g_j\mu_N B\hat{I}^j_x + \frac{h\nu_{\text{Q}}}{6}\left(3\hat{I}^{j\,2}_{z'} - \hat{I}^{j\,2} + \eta\left(\hat{I}^{j\,2}_{x'} - \hat{I}^{j\,2}_{y'}\right)\right). \tag{3}$$

Here, (x, y, z) is the coordinate set in the laboratory frame and (x', y', z') denotes the frame of the quadrupolar field, which is specific to the location of the nucleus "j". We reference the orientation of the quadrupolar frame relative to the lab using the Euler angles (φ, θ, ψ). g_j is the g-factor of the nucleus, μ_N is the nuclear magneton, B is the external magnetic field, ν_{Q} is the characteristic energy for the quadrupolar interaction the location of the nucleus "j" and η is the biaxiality parameter.

Nuclear Spin correlator

The spin-correlator for the nucleus "j", along the axis $\alpha = x, y, z$ reads:

$$\langle \hat{I}^j_\alpha(\tau)\hat{I}^j_\alpha(0)\rangle = tr\left(e^{+\frac{i\hat{H}^j_{\text{nuc}}\tau}{\hbar}}\hat{I}^j_\alpha e^{-\frac{i\hat{H}^j_{\text{nuc}}\tau}{\hbar}}\hat{I}^j_\alpha\rho\right). \tag{4}$$

If we take the infinite temperature density matrix $\rho = \mathbb{I}/(2I+1)$, and if we consider the eigenvalues and eigenvectors $\left\{\hbar\omega^j_l, |\psi^j_l\rangle\right\}_l$ of H^j_{nuc}, the spin-correlator can formally be written:

$$\langle \hat{I}_\alpha^j(\tau)\hat{I}_\alpha^j(0)\rangle = 2\sum_{l=1}^{2I+1}\sum_{m>l}^{2I+1}\frac{\left|\langle\psi_m^j|\hat{I}_\alpha^j|\psi_l^j\rangle\right|^2}{2I+1}cos\left(\omega_{lm}^j\tau\right) + \sum_{l=1}^{2I+1}\frac{\left|\langle\psi_l^j|\hat{I}_\alpha^j|\psi_l^j\rangle\right|^2}{2I+1}, \quad (5)$$

with $\omega_{lm}^j = \left|\omega_l^j - \omega_m^j\right|$.

We emphasize that "j" designates a given set of parameters $\{(\varphi, \theta, \psi), \nu_\mathrm{Q}, \eta\}$. The nuclear spin correlator can be decomposed into (i) a time-invariant contribution and (ii) a time-varying contribution. In the time-varying contribution, the frequencies correspond to the nuclear Larmor frequency or multiples thereof. The time-invariant contribution is strictly at zero frequency in our model. Knowing the quasi-static noise amplitude from the FID decay, an agreement between the spin-echo data at long delays and the model requires a bin-size smaller than 1 kHz which constitutes an upper bound for the quasi-static noise frequency spread. Physically, this contribution describes the 'frozen' nuclear spin bath evolving on very slow timescales due to nuclear-nuclear interactions, and is the main cause for the electron FID discussed earlier.

We then generalise the single spin description to a correlator for the nuclear isotope "k", denoted $R_\alpha^k(\tau)$, by averaging over a Gaussian probability distribution for the quadrupolar field amplitude (ν_Q) and angular distribution (θ). φ and ψ are varied from 0 to 2π in steps of $\pi/2$, which corresponds to the external magnetic field being aligned with the $[110]$ or $[1\bar{1}0]$ crystal axis:

$$R_\alpha^k(\tau) = \frac{1}{16}\sum_{\varphi,\psi}\iint d\theta d\nu_\mathrm{Q}\frac{e^{-\frac{(\theta-\theta_k)^2}{2\sigma_{\theta_k}{}^2}-\frac{\left(\nu_\mathrm{Q}-\nu_{\mathrm{Q}_k}\right)^2}{2\sigma_{\nu_{\mathrm{Q}_k}}{}^2}}}{\left(\sqrt{2\pi}\right)^2\sigma_{\theta_k}\sigma_{\nu_{\mathrm{Q}_k}}}\langle \hat{I}_\alpha^j(\tau)\hat{I}_\alpha^j(0)\rangle\,. \quad (6)$$

The spin-correlator taken at $\tau = 0$ is related to the variance of the nuclear field:

$$\sigma^2(\boldsymbol{B}_\mathrm{OH}) = \frac{2}{\left(g_e\mu_B\sqrt{N}\right)^2}\sum_{k,\alpha}x_k A_k{}^2 R_\alpha^k(0). \quad (7)$$

This relation will be used to weight the noise power spectra introduced in the next paragraph.

Noise spectrum due to nuclear precession

Due to the large difference between electron and nuclei Zeeman energies, their dynamics can be decoupled, and to first order, the electron precesses around a total magnetic field $\boldsymbol{B}_{\text{tot}} = \boldsymbol{B}_{\text{ext}} + \boldsymbol{B}_{\text{OH}}$. This semi-classical picture, originally introduced in Ref. 2, predicts a modification of the electronic level splitting by the Overhauser field following:

$$\Delta E_{\text{Zeeman}}^{\text{electron}} = g_e \mu_B \|\boldsymbol{B}_{\text{tot}}\|$$

$$\approx g_e \mu_B \left(B_{\text{ext}} + B_{\text{OH}x} + \frac{B_{\text{OH}y}^2 + B_{\text{OHz}}^2}{2B_{\text{ext}}} \right).$$

The noise spectrum which affects the electron coherence can be decomposed into three power spectral densities: $\mathcal{S}_{\parallel}^{0}(\omega)$ which is a delta function at 0-frequency, with an amplitude controlled by the constant terms in Equation (5) and $\mathcal{S}_{\parallel}(\omega)$ and $\mathcal{S}_{\perp}(\omega)$ which describe the first-order coupling of nuclear fluctuations along the B-field axis and the second-order coupling of nuclear fluctuations perpendicular to the B-field, respectively.

$\mathcal{S}_{\parallel}^{0}(\omega)$ and $\mathcal{S}_{\parallel}(\omega)$ are respectively given by

$$\mathcal{S}_{\parallel}^{0}(\omega) + \mathcal{S}_{\parallel}(\omega) = \left(\frac{1}{\hbar^2 N}\right) \sum_k x_k {A_k}^2 \, s_x^k(\omega)\,, \tag{8}$$

where $s_x^k(\omega)$ represents the Fourier transform of the spin-correlator for the species k introduced earlier:

$$s_x^k(\omega) = \int d\tau \, e^{-i\omega\tau} \, R_x^k(\tau)\,. \tag{9}$$

Using equations (5)-(9) and the fact that $\int d\tau \, e^{-i\omega\tau} \, cos\left(\omega_{lm}^{j}\tau\right) = \pi \, \left(\delta\left(\omega - \omega_{lm}^{j}\right) + \delta\left(\omega + \omega_{lm}^{j}\right)\right)$, we obtain:

$$\mathcal{S}_{\parallel}^{0}(\omega) = \left(\frac{1}{\hbar^2 N}\right) \sum_k x_k {A_k}^2 \sum_{\varphi,\psi} \iint d\theta d\upsilon_{\text{Q}} \frac{e^{-\frac{(\theta-\theta_k)^2}{2{\sigma_{\theta_k}}^2} - \frac{\left(\nu_{\text{Q}}-\nu_{\text{Q}_k}\right)^2}{2{\sigma_{\nu_{\text{Q}_k}}}^2}}}{16 \ \sigma_{\theta_k}\sigma_{\nu_{\text{Q}_k}}} \times \sum_{l=1}^{2I_k+1} \frac{\left|\left\langle \psi_l^j \middle| I_x^j \middle| \psi_l^j \right\rangle\right|^2}{2I_k + 1} \delta(\omega), \tag{10}$$

$$\mathcal{S}_{\parallel}(\omega) = \left(\frac{1}{\hbar^2 N}\right) \sum_k x_k {A_k}^2 \sum_{\varphi,\psi} \iint d\theta d\upsilon_{\mathrm{Q}} \frac{e^{-\frac{(\theta-\theta_k)^2}{2{\sigma_{\theta_k}}^2}-\frac{\left(\nu_{\mathrm{Q}}-\nu_{\mathrm{Q}_k}\right)^2}{2{\sigma_{\nu_{\mathrm{Q}_k}}}^2}}}{16\ \sigma_{\theta_k}\sigma_{\nu_{\mathrm{Q}_k}}} \times \sum_{l=1}^{2I_k+1} \sum_{m>l}^{2I_k+1} \frac{\left|\langle \psi_m^j | I_x^j | \psi_l^j \rangle\right|^2}{2I_k+1} \left(\delta\left(\omega-\omega_{lm}^j\right)+\delta\left(\omega+\omega_{lm}^j\right)\right). \tag{11}$$

From equation (11), we see that the frequency components in the noise spectrum $\mathcal{S}_{\parallel}$ correspond to the Larmor frequencies of the different nuclear species. The amplitude of this noise depends on the matrix element $\langle \psi_m^j | I_x^j | \psi_l^j \rangle$. In the absence of quadrupolar coupling, this matrix element is null for $m \neq l$ as I_x^j commutes with the Hamiltonian $\widehat{H}_{\mathrm{nuc}}^j$. It is only the presence of quadrupolar coupling, along an axis which differs from the magnetic field that allows $\mathcal{S}_{\parallel}(\omega)$ to differ from 0. The corresponding physical picture is that the quadrupolar field adds to the external field to create a precession which is tilted from $\boldsymbol{B}_{\mathrm{ext}}$. As this simple picture suggests, the amplitude of the first-order coupling will decrease with increasing magnetic field, as the quadrupolar term becomes negligible compared to the Zeeman term. We note, however, that a different mechanism of first order coupling has recently been reported by Botzem *et al.* in gate-defined quantum dots[2]. In this case, the coupling is due to an anisotropy of the electron g-factor. Interestingly, this leads to a noise amplitude which is independent from the external B-field.

To calculate $\mathcal{S}_{\perp}(\omega)$, it is convenient to use a convolution in the spectral domain. We find:

$$\mathcal{S}_{\perp}(\omega) = \frac{1}{(2g_e\mu_B B_{\mathrm{ext}})^2}\left(\frac{1}{\hbar^2 N}\right)^2 \times \frac{1}{2\pi}\int d\omega' \sum_{\alpha=y,z}\sum_{k,k'} x_k {A_k}^2 x_{k'} {A_{k'}}^2\, s_\alpha^k(\omega) s_\alpha^{k'}(\omega'-\omega)\,. \tag{12}$$

The quadratic coupling to the transverse Overhauser field results in an auto-convolution in the spectral domain. This causes the broad-frequency shoulder around zero frequency that is so detrimental to the electron spin coherence.

Delay-dependence of the Hahn echo and the FID

Once the noise spectrum has been calculated, the Hahn echo or the FID can be computed using the filter function corresponding to the echo pulse sequence. For example, this approach is presented in detail in Ref. 1. Underlying the model is the fact that both linearly

and quadratically coupled Overhauser field components can be treated as Gaussian noise. For the quadratic component, such an assumption is justified in the case of InGaAs quantum dots because the noise correlation time is shorter than the time T at which the spin-echo coherence is retrieved[3]. Following the notations from Ref. 1, the decoherence function $W(t)$ is expressed as:

$$W(t) = W_0 \, e^{-\chi(t)} \, , \tag{13}$$

where W_0 is the maximum visibility, limited by pulse imperfections, and the function $\chi(t)$ is expressed as a function of the total noise spectrum $\mathcal{S}_{\text{tot}}(\omega)$:

$$\chi_{HE}(t) = \int_0^\infty \frac{d\omega}{\pi} \mathcal{S}_{\text{tot}}(\omega) \frac{8 \sin^4(\omega t/4)}{\omega^2}, \tag{14}$$

and

$$\chi_{FID}(t) = \int_0^\infty \frac{d\omega}{\pi} \mathcal{S}_{\text{tot}}(\omega) \frac{2 \sin^2(\omega t/2)}{\omega^2}. \tag{15}$$

Model parameters

We base our mean-field calculation of the nuclear spin spectra, and hence the electron Hahn echo, on mean parameters found in Bulutay's atomistic calculation[4,5]. The indium concentration determines overall strain characteristics, affecting all relevant parameters. We perform a least-mean-squared analysis of the calculated spin echo to our data and find best agreement for an indium concentration between 0.4 and 0.7. All calculations in this paper use $x_{In} = 0.5$ and the parameters in Supplementary Table 2.

We note a sum of two Gaussian distributions is used to describe θ_{In}. The relevant weights w_i are $w_1 = 0.55$ and $w_1 = 0.45$. Further, to account for a mismatch of the direction of the applied magnetic field and the sample edges (along the $[110]$ and $[1\bar{1}0]$ crystal directions) the angle φ is offset by 10 ° from zero.

Spectra of nuclear spin species

Supplementary Figure 3 highlights the composition of the perpendicular and parallel spin spectra in terms of the four nuclear species for two magnetic fields. Indium plays the dominant part due to its large spin quantum number, followed by arsenic, which contributes the majority of atoms to the quantum dot. Gallium nuclei play a negligible part overall.

Sub-kHz narrow linewidths have been found in recent nuclear magnetic resonance measurements on individual neutral quantum dots[6]. While we expect significantly broadened linewidths for charged quantum dots due to hyperfine-mediated nuclear spin-spin interaction we find a negligible effect on the nuclear spectra for linewidths below 100 kHz, where quadrupolar broadening masks any effects of nuclear spin coherence.

Effect of linear and quadratic coupling of $\boldsymbol{B}_{\mathbf{OH}}$ on spin coherence and magnetic field dependence

To provide some intuition on the role of the first- and second-order coupling to the nuclear spin dynamics we show deconstructed spin echo functions from Fig. 3, main text, in Supplementary Figure 4. Here, the two terms in the noise spectrum have been used individually to compute $\chi(t)$ as input to the decoherence function $W(t)$.

As outlined in the main text, the linearly coupled parallel component of $\boldsymbol{B}_{\mathbf{OH}}$ is almost entirely responsible for the modulation of the spin echo signal. The collapse and revival of the spin coherence is strongly damped due to the complexity of the nuclear spin spectrum, which arises from both magnitude and inhomogeneity of the nuclear quadrupolar interaction. The perpendicular component of $\boldsymbol{B}_{\mathbf{OH}}$ couples to second order to the electron spin and provides a mostly smooth envelope to the echo function.

These Hahn-echo fitting coefficients correspond to an Overhauser field standard deviation ($\sigma(\boldsymbol{B}_{\mathrm{OH}})$) of 40 mT for $\mathcal{S}_{\parallel}(\omega)$ and 28 mT for $\mathcal{S}_{\perp}(\omega)$. This is to be compared with a 33 mT standard deviation for $\mathcal{S}_{\parallel}^{0}(\omega)$ that we used to reproduce the FID decay with the same spectra but with a different filter function. The functional form of the decay approaches a $e^{-(t/T_2)^4}$ dependence as the magnetic field tends to zero. Interestingly, Press *et al*. reported a very similar magnetic field dependence in the range between 2 and 4 T [7], followed by a saturation

of coherence at higher fields. Lifetime limitations are expected to play a role only at the 10-T level and beyond[8,9].

We do find the Hahn echo coherence at high magnetic fields to be very sensitive to experimental imperfections. Imperfect suppression of the readout laser during the long free evolution of the electron spin, for example, causes spin pumping which is equivalent to a T_1 decay.

In comparison to the Hahn-echo data in Refs. 7 and 10, the pickup of spin coherence in our quantum dot sample takes place at a higher magnetic field and consequently coherence times at low and intermediate fields are shorter. In particular, in the low-field limit coherence is lost within ~ 20 ns in our case, while a similar measurement gives ~ 30 ns in Ref. 10. This points to a higher indium concentration and consequently a larger strain in our sample. We note that the Hahn-echo amplitude is rescaled in Refs. 7 and 10 while we use the measured fringe visibility. The normalisation has to be taken into account for a quantitative comparison. Drawing quantitative conclusions about the structural composition of the quantum dots in Refs. 7 and 10 based on our measurements alone is challenging due to the complexity of the nuclear spin bath. This may be possible with nuclear bath spectra calculated from atomistic models, such as the ones of Bulutay [4,5]. With this caveat in mind, our model predicts that by reducing the indium content slightly (x=0.4), accompanied with the expected reduction in quadrupolar field strength (following Bulutay, Ref. 4) and an equivalent reduction in the inhomogeneity, the transition to high spin coherence takes place at a magnetic field of ~ 2 T instead of 3 T, and spin coherence at 4 T increases from ~1 μs to 1.5 μs. This is in line with the qualitative expectation that lower and more homogeneous strain reduces the effects of the second-order contributions of the hyperfine interaction.

Amplitude of Hahn-echo measurements

The amplitude in our Hahn-echo measurements corresponds to the visibility in the readout signal oscillations as the delay of the central pulse is scanned over several Larmor periods. The sub-unity visibility at short storage times T is due to a non-equatorial rotation axis which prevents a complete spin inversion with the π -pulse. A good estimate of the inversion fidelity can be found with the pulse scheme used in Fig. 3 (a, b) of the main text: the ratio of the readout visibility for the second half, where the electron spin is (imperfectly) inverted before the next $\pi/2$ -pulse and the readout visibility obtained for the first half of the pulse

sequence is a measure of the inversion fidelity. With increasing magnetic fields, the Larmor rotation during the picosecond pulse increases, yielding a lower inversion fidelity.

Supplementary References

1. Cywiński, L., Lutchyn, R. M., Nave, C. P. & Das Sarma, S. How to enhance dephasing time in superconducting qubits. *Phys. Rev. B* **77,** 174509 (2008).
2. Botzem, T. *et al.* Quadrupolar and anisotropy effects on dephasing in two-electron spin qubits in GaAs. *Nat. Commun.* **7,** 11170 (2016).
3. Cywiński, Ł. Dynamical-decoupling noise spectroscopy at an optimal working point of a qubit. *Phys. Rev. A* **90,** 42307 (2014).
4. Bulutay, C. Quadrupolar spectra of nuclear spins in strained In x Ga 1 − x As quantum dots. *Phys. Rev. B* **85,** 115313 (2012).
5. Bulutay, C., Chekhovich, E. A. & Tartakovskii, A. I. Nuclear magnetic resonance inverse spectra of InGaAs quantum dots: Atomistic level structural information. *Phys. Rev. B* **90,** 205425 (2014).
6. Waeber, A. M. *et al.* Few-second-long correlation times in a quantum dot nuclear spin bath probed by frequency-comb nuclear magnetic resonance spectroscopy. *Nat. Phys.* (2016). doi:10.1038/nphys3686
7. Press, D. *et al.* Ultrafast optical spin echo in a single quantum dot. *Nat. Photonics* **4,** 367–370 (2010).
8. Lu, C.-Y. *et al.* Direct measurement of spin dynamics in InAs/GaAs quantum dots using time-resolved resonance fluorescence. *Phys. Rev. B* **81,** 35332 (2010).
9. Kroutvar, M. *et al.* Optically programmable electron spin memory using semiconductor quantum dots. *Nature* **432,** 81–84 (2004).
10. Bechtold, A. *et al.* Three-stage decoherence dynamics of an electron spin qubit in an optically active quantum dot. *Nat. Phys.* **11,** 1005–1008 (2015).
